# Development and validation of a multimodal feature fusion-based model for predicting postoperative recurrence-free survival in locally advanced laryngeal squamous cell carcinoma

**DOI:** 10.3389/fonc.2025.1685737

**Published:** 2025-09-25

**Authors:** Feng Zhao, Xiaoying Huang, Jiangmiao Li, Junkun He, Jiaxin Liu, Guanwei Chen, Zhe Zhang

**Affiliations:** Department of Otolaryngology, Head and Neck Surgery, The First Affiliated Hospital of Guangxi Medical University, Nanning, China

**Keywords:** laryngeal squamous cell carcinoma, locally advanced, multimodal features, recurrence-free survival, decision-level fusion

## Abstract

**Objectives:**

Given the high postoperative recurrence of locally advanced laryngeal squamous cell carcinoma (LSCC) and American Joint Committee on Cancer (AJCC) staging system prediction limitations, this study aims to construct and validate a postoperative recurrence-free survival (RFS) prediction model using multimodal feature fusion and explore data integration strategies to enhance prediction efficacy.

**Methods:**

Data from 278 patients diagnosed with locally advanced LSCC between 2013 and 2024 were collected retrospectively. These data were then separated into a training dataset (n = 196) and a validation dataset (n = 82), using a near 7:3 allocation strategy. By integrating clinicopathological features, preoperative blood markers, and enhanced computed tomography imaging data, we constructed clinicopathological (Clinic-score), radiomics (Rad-score), and two fusion models: feature-level (FF-Model) and decision-level (DF-Model). Model performance was evaluated using the concordance index, time-dependent area under the receiver operating characteristic curve, calibration curve, and decision curve analyses. Improvement in model discriminative ability was assessed using continuous net reclassification improvement (cNRI) and integrated discrimination improvement (IDI).

**Results:**

At 24.5 months median follow-up, 95 patients (34.2%) experienced recurrence. In the validation set, the DF-Model significantly outperformed the FF-Model, Rad-score and Clinic-score models, and AJCC stages. Additionally, the DF-Model demonstrated superior calibration and clinical utility, better prediction of 1-year, 3-year, and 5-year RFS through cNRI/IDI analysis, and excellent risk stratification across datasets, AJCC stages, and tumor locations.

**Conclusion:**

The multimodal prediction DF-Model effectively integrates multi-source heterogeneous information, significantly improving the prediction accuracy of postoperative RFS in locally advanced LSCC, outperforming the FF-Model, single-modal models, and AJCC staging system, and demonstrating its potential clinical translational value.

## 1 Introduction

Laryngeal squamous cell carcinoma (LSCC), one of the most common malignant tumors of the head and neck region, is increasing in incidence among males annually, accounting for most of the approximately 180,000 new cases of laryngeal cancer globally each year (1, 2). Notably, approximately 43.1–65% of patients are diagnosed with locally advanced stage (III, IVa, and IVb) disease at initial presentation, resulting in a five-year disease-free survival rate of only 50–65% (3–7). Despite the widespread application of comprehensive treatment strategies, including surgery, radiotherapy, and chemotherapy, approximately 30–40% of patients experience local tumor recurrence or distant metastatic spread after surgery, which can substantially compromise long-term survival and quality of life (4, 7, 8).

Currently, clinical practice relies primarily on the TNM staging system of the American Joint Committee on Cancer (AJCC) for prognostic assessment. However, its reliance on anatomical criteria overlooks critical biological and systemic factors—such as tumor heterogeneity, host systemic status, and treatment quality—that significantly influence outcomes. Consequently, its predictive accuracy for recurrence, as measured by the concordance index (C-index), is suboptimal (C-index<0.65), limiting the utility of precise individualized risk stratification (9, 10). In addition, systemic inflammatory indicators (such as the neutrophil-to-lymphocyte ratio (NLR), platelet-to-lymphocyte ratio (PLR), and lymphocyte-to-monocyte ratio (LMR)) and nutritional prognostic indices (such as the prognostic nutritional index (PNI)) have attracted considerable attention; however, the predictive efficacy of single markers is limited and susceptible to various factors (11, 12). Novel molecular markers (such as epidermal growth factor receptor overexpression (13), WRAP53β (14), sex hormone receptors estrogen receptor-β and progesterone receptor (15), and p53 mutation (16)) have shown significant prognostic value; however, their clinical application requires further validation.

With advancements in artificial intelligence (AI) and precision medicine, the synergistic use of diverse data modalities—such as clinical, imaging, and molecular profiles—combined with AI algorithms—has shown great promise in enhancing prognosis prediction. In areas such as high-grade serous ovarian cancer (17), glioblastoma (18), thyroid cancer (19), renal cell carcinoma (20), breast cancer (21), and colorectal cancer (22), the area under the receiver operating characteristic (ROC) curve (AUC) values and C-indices (AUC > 0.8; C-index > 0.7) of multimodal data fusion models generally outperform traditional methods. However, research on predicting the risk of postoperative recurrence in locally advanced LSCC still has limitations, most of which are confined to application scenarios of a single type or single-modality data source, such as relying solely on clinicopathological features (23) or radiomics parameters (24–26), with AUC values generally<0.8, making it difficult to meet the need for precise stratification of patients.

Therefore, this study aimed to develop a comprehensive prediction model for postoperative recurrence-free survival (RFS) in locally advanced LSCC by synthesizing preoperative data from multiple domains—clinicopathological, laboratory, and radiomic—thereby leveraging the strengths of multimodal fusion. By comparing the efficacy of feature- and decision-level fusion strategies, this study sought to achieve precise stratification of postoperative recurrence risk, thereby providing data support for the formulation of personalized treatment plans (such as adjuvant radiotherapy dose adjustment) and dynamic prognosis management. We compared feature- and decision-level fusion because they represent fundamentally different integration strategies: the former concatenates features early (potentially capturing interactions but risking overfitting), while the latter combines model predictions, preserving modality-specific patterns and enhancing interpretability. This comparison helps identify the optimal architecture for clinical prediction in LSCC.

## 2 Materials and methods

### 2.1 Study participants

Following the principles of the TRIPOD-AI checklist (27), we conducted a retrospective cohort study of patients with locally advanced LSCC at the First Affiliated Hospital of Guangxi Medical University (Jan 2013–Jan 2024). Inclusion was determined by histopathologic staging per AJCC 8th edition (Stage III–IVb) (9), availability of more than six months of postoperative monitoring, documented results for standard blood counts as well as liver and kidney function indicators, and qualified preoperative contrast-enhanced multiplex spiral computed tomography (CT) scan images. The exclusion criteria were incomplete medical records; history of surgery, radiotherapy, or chemotherapy prior to surgery; presence of active infections, chronic inflammation, hematological diseases, or autoimmune diseases; and history of other malignancies.

**Figure 1:**
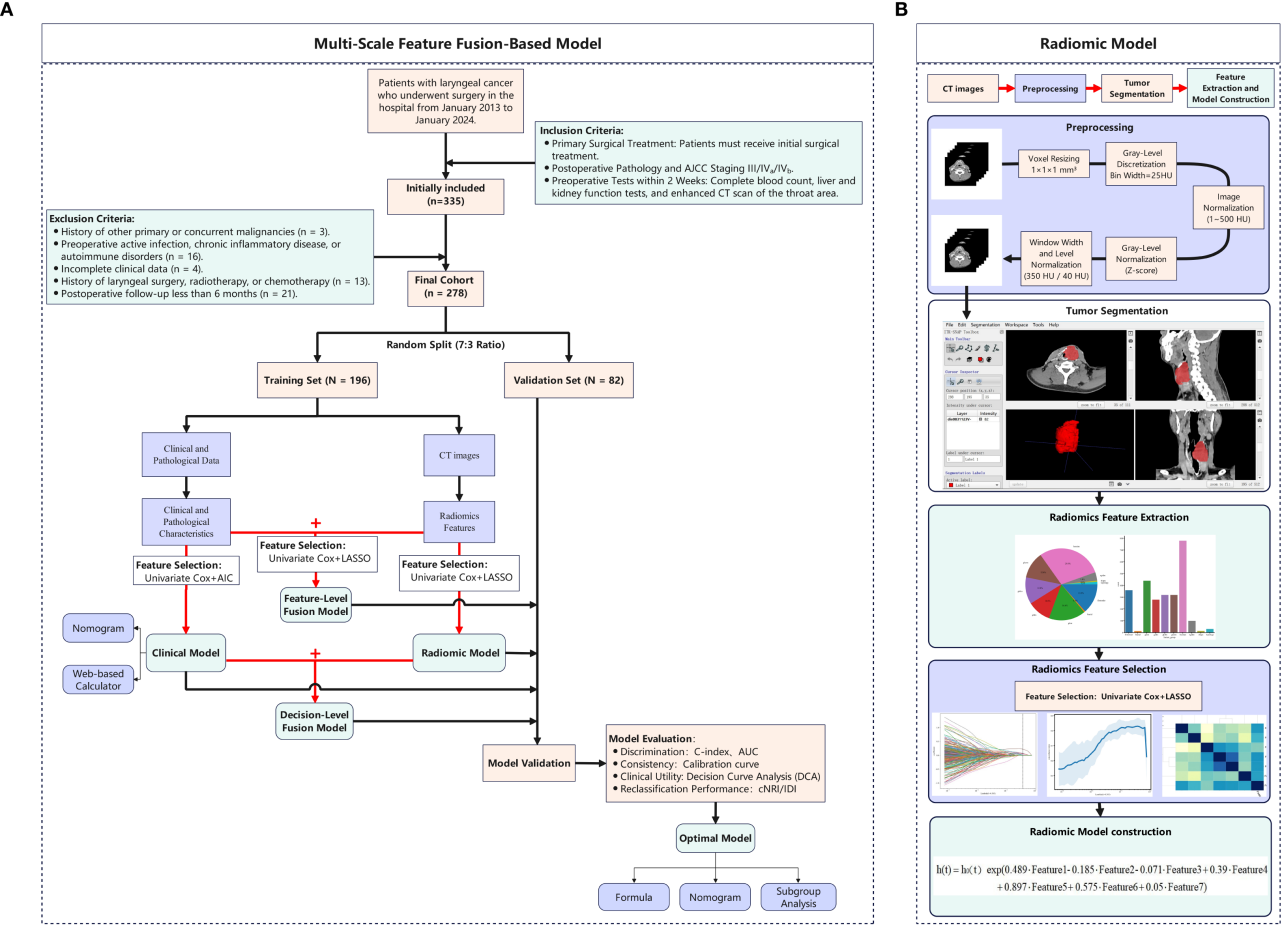
Methodological framework of the study design. **(A)** Multi-Scale Feature Fusion-Based Model; **(B)** Radiomic Model.

The sample size was calculated using the Events Per Variable (EPV) method: N = (EPV × number of predictor variables/recurrence rate) × (1 + efficiency rate). With 7 expected variables, a 30% 5-year recurrence rate, EPV = 10, and 10% inefficiency, a minimum of 256 patients was required. After applying criteria, 278 patients were included, meeting statistical power needs. The study adhered to the Declaration of Helsinki and was approved by the Institutional Ethics Committee (No. 2025-E0564). Informed consent was waived for this retrospective study, and all data were anonymized. The study workflow is shown in **Figure 1**.

### 2.2 Acquisition of clinical variables

Clinical data were extracted from electronic health records, including age, sex, comorbidities (e.g., hypertension), smoking/alcohol history, preoperative blood count, liver/kidney function, and postoperative pathological features (tumor location, margin status, vascular invasion, lymph node metastasis, differentiation, and TNM stage). Based on preoperative peripheral blood test results, we calculated the following systemic inflammation-related biomarkers: NLR, PLR, LMR, Systemic Immune-Inflammation Index (SII) = platelet count × NLR, PNI = lymphocyte count × 5 + albumin, Advanced Lung Cancer Inflammation Index (ALI) = body mass index (BMI) × albumin/NLR, and Systemic Inflammation Response Index (SIRI) = monocyte count × NLR. Blood tests used the Beckman Coulter/LH 780 and Werfen/ACL TOP 750LAS, with collection between 6:00 and 10:00 AM. Smoking history was defined as >1 cigarette/day for >6 months pre-surgery; drinking history as ≥72g alcohol/week for >6 months pre-surgery. The AJCC 8th edition (2017) criteria were applied for pathological staging.

### 2.3 Image acquisition and preprocessing

Contrast-enhanced CT scans (skull base to supraclavicular) were performed. Venous phase images were acquired 60–90 s after injecting non-ionic contrast (iopromide/iohexol, 350 mg/ml, 1.0–2.0 ml/kg, 3–5 ml/s). Scanner parameters are in **Supplementary Table 1**. Image preprocessing followed the Evaluation of Radiomics Research (CLEAR) checklist (28): original DICOM images were resampled to 1×1×1 mm³ isotropic resolution, gray-level discretized (bin width: 25 HU), intensity range limited to 1–500 HU, and normalized using Z-score. Window width/level was set to 350/40 HU to enhance tumor boundaries. The tumor volume of interest (VOI) was outlined at the beginning of the study by randomly selecting CT images from 35 patients. The delineation of tumor boundaries on axial images was conducted independently by two head and neck specialists, who had accumulated 10 and 13 years of clinical expertise, using the ITK-SNAP platform (www.itksnap.org) without knowledge of other clinical data (**Figure 1B**). One week later, the more experienced rater repeated the segmentation. Inter-rater agreement was assessed using the intraclass correlation coefficient (ICC). Features with ICC > 0.75 were retained for analysis.

### 2.4 Prediction of outcomes and follow-up

RFS was defined as time from surgery to the first recurrence or metastasis or the end of follow-up (Jan 31, 2025), with time points set at 1, 3, and 5 years postoperatively. Follow-up used digital communication (WeChat), telephonic contact, and clinic visits (quarterly for years 1–3, semi-annually for years 4–5, annually thereafter), with<5% loss to follow-up.

### 2.5 Model development and validation

#### 2.5.1 Dataset partitioning

To ensure reproducibility, we split the dataset into two subsets — a training set (n = 196) and a validation set (n = 82) — using a 7:3 partitioning strategy with a fixed random seed (seed = 42). The training set was used to train and optimize the model, whereas the validation set was used to evaluate the generalization ability of the model.

#### 2.5.2 Development of clinical-pathological model (Clinic-score)

Based on 35 baseline clinicopathological characteristics and peripheral blood markers of patients in the training set, variables were screened using univariate Cox regression (p< 0.05), and the optimal variable combination was selected in conjunction with the Akaike Information Criterion (AIC) (29) to construct a multivariate Cox regression model, which was defined as the clinical-pathological model (Clinic-score). The results are presented as a nomogram, and a web-based calculator was developed.

#### 2.5.3 Development of radiomics models (Rad-score)

Radiomics features were extracted from preprocessed tumor VOIs in the training cohort using PyRadiomics, encompassing ten feature classes: first-order statistics, 3D morphological features, gray-level co-occurrence matrix (GLCM), gray-level size zone matrix (GLSZM), gray-level run length matrix (GLRLM), neighborhood gray-tone difference matrix (NGTDM), Hessian-based features, fractal features, and topological features. Univariate Cox regression (P< 0.05) identified prognostic features, which were further refined using the least absolute shrinkage and selection operator (LASSO) with 10-fold cross-validation to minimize overfitting. The optimal regularization parameter (λ) was selected by minimizing cross-validated partial likelihood deviance, retaining only features with non-zero coefficients. A multivariable Cox model was then fitted to these features to compute the radiomics score (Rad-score):


$$\text{Rad}-\text{score}=\sum_{i=1}^{k}\beta_{i}\;X_{i}$$

Where $X_{i}$ is the value of the i-th radiomics feature, $\beta_{i}$ is its corresponding regression coefficient from the multivariable Cox model, and k is the number of selected features.

#### 2.5.4 Development of fusion models

We compared feature-level and decision-level fusion strategies to determine the optimal approach for integrating multimodal data (clinical, blood, and radiomics). Feature-level fusion combines raw features, while decision-level fusion integrates model outputs, offering potentially greater robustness.

*Feature-level fusion model (FF-Model)*

1. Variable selection: This model involves the direct concatenation of clinicopathological features, peripheral blood markers, and radiomics features, which are selected through univariate Cox regression during the construction of the radiomics model. Subsequently, a LASSO-Cox regression approach incorporating 100 rounds of 10-fold cross-validation is applied to select relevant predictors.

2. Fusion strategy: Early fusion — raw features from different modalities are concatenated into a single vector.

3. Model construction: A multivariable survival model is constructed based on Cox regression. The resulting linear predictor is defined as the FF-Score.

*Decision-level fusion model (DF-Model)*

1. Variable selection: This model uses the outputs of two pre-trained submodels as input variables:

(i) Clinic-score: derived from the clinical-pathological model (Section 2.5.2);

(ii) Rad-score: derived from the radiomics model (Section 2.5.3).

2. Fusion strategy: Late fusion — predictions (scores) from separate submodels are combined at the decision level.

Model construction: The Clinic-score and Rad-score are combined in a multivariable Cox proportional hazards model as covariates. The model is defined as: $h(t)=h_{0}(t)\exp(\beta_{1}\cdot Clinic-score+\beta_{2}\cdot Rad-score)$ . Where *h_0_(t)* is the baseline hazard, and *β_1_*, *β_2_* are the maximum likelihood estimates for the respective regression coefficients.

#### 2.5.5 Model evaluation and comparison

Model performance was evaluated based on discrimination, calibration, and clinical utility. Discrimination was assessed using the C-statistic and time-dependent AUC at 1, 3, and 5 years for RFS. Calibration was evaluated using calibration plots. Clinical utility was assessed via decision curve analysis (DCA), comparing models against each other and the AJCC 8th Edition TNM staging system. Additionally, the degree of improvement in the fusion model was assessed using continuous net reclassification improvement (cNRI) and integrated discrimination improvement (IDI). Finally, the optimal cutoff for the best-performing model was determined using X-tile software (30), stratified by AJCC stage and tumor location, to validate its risk stratification and generalizability across subgroups.

### 2.6 Statistical methods

Categorical variables (such as tumor location), are summarized with counts and proportions, while group differences were assessed using either the chi-square test or Fisher’s exact test. For continuous variables (such as NLR), the data are expressed as mean ± standard deviation (mean ± SD) or median and interquartile range (IQR), contingent upon whether the data adhered to a normal distribution. An independent samples t-test or Mann–Whitney U test was used to evaluate differences between groups. The statistical processing was carried out using R version 4.2.3 and Python 3.6, with key functions drawn from these packages: glmnet 4.1.8, pROC 1.18.5, rms 6.7.1, dplyr 1.1.4, survival 3.7.0, and timeROC 0.4. Statistical significance was set at p< 0.05.

## 3 Results

### 3.1 Patient characteristics

A total of 278 patients with locally advanced LSCC were enrolled in this study, including 106 (38.1%) patients with stage III, 170 (61.1%) with stage IVa, and two (0.7%) with stage IVb disease. The median follow-up duration was 24.5 (8.3–54.8) months. As of the follow-up cutoff date of January 31, 2025, 95 (34.2%) patients experienced recurrence or distant metastasis. The median time from the last treatment to recurrence or metastasis was 10 (6–21) months. The cumulative RFS rates at 1, 3, and 5 years were 79.5%, 70.1%, and 67.3%, respectively. The cumulative recurrence rate was 35.2% (69/196) in the training set and 31.7% (26/82) in the validation set, with no significant differences between the recurrence rates of the two sets (p > 0.05). Except for BMI (Z = 2.204, p = 0.028) and smoking history (χ² = 3.884, p = 0.049), there were no significant differences in the distribution of the remaining variables between the training and validation sets (all p > 0.05, **Supplementary Table 2**).

### 3.2 Clinic-score

**Figure 2:**
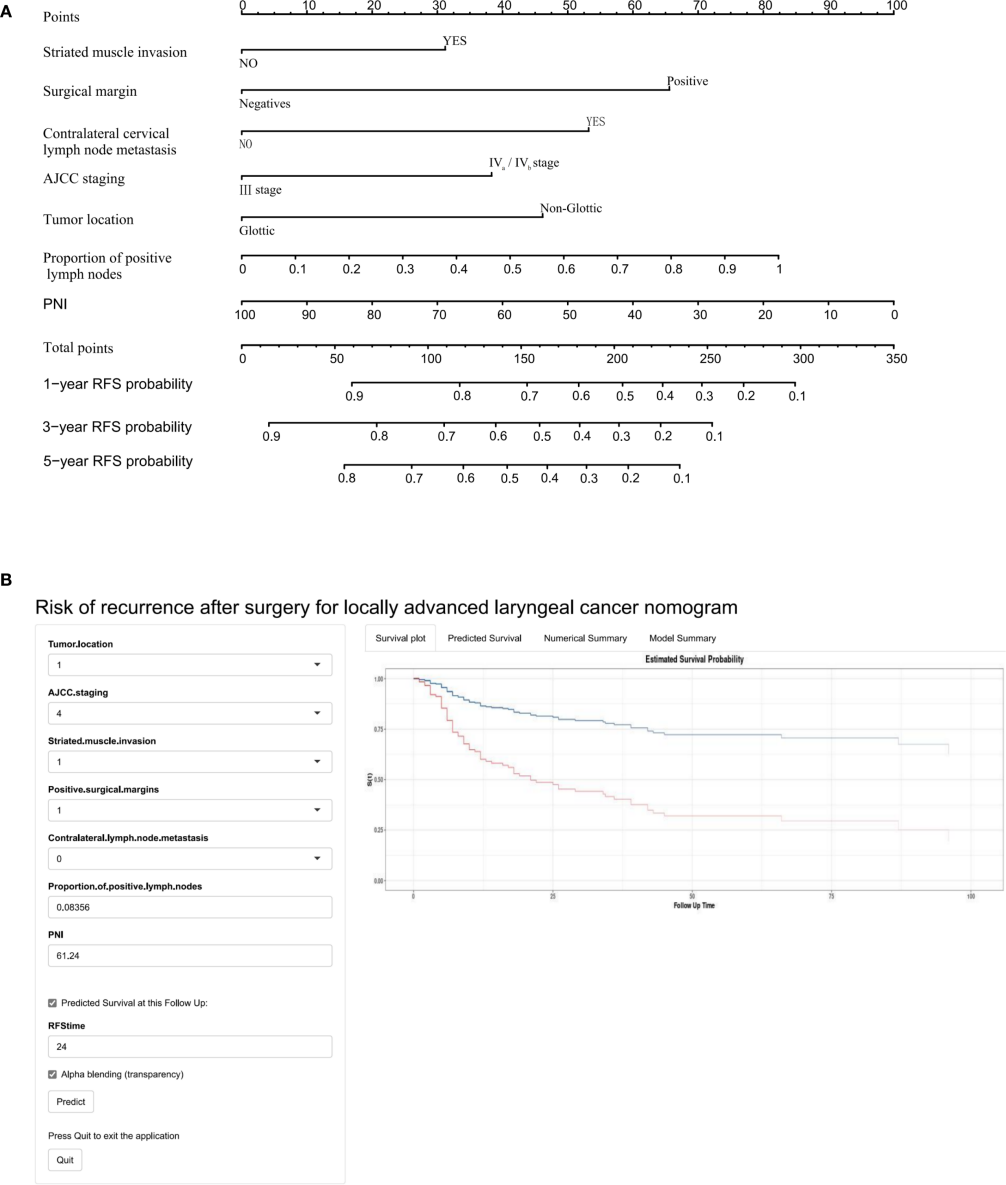
A clinicopathological model for predicting RFS after surgery in locally advanced LSCC. **(A)** Nomogram, **(B)** Web-based calculator.

Univariate Cox regression analysis identified 13 potential predictive variables (including tumor location and AJCC stage; all p< 0.05; **Table 1**). Based on the AIC, seven optimal variables were determined to construct the multivariate Cox regression model (**Supplementary Table 3**). Additionally, the model was visualized as a nomogram and deployed a web calculator at https://huangxiaoying.shinyapps.io/dynnomapp (**Figure 2**).

**Table 1:** Univariate Cox regression analysis of postoperative RFS in patients with locally advanced LSCC.

Variables	Beta	Standard error	Z-value	HR(95%CI)	p-value
Age,year	0.018	0.013	1.398	1.018(0.993,1.045)	0.162
Sex,n(%)					
Male				Reference	
Female	-0.791	1.008	0.785	0.453(0.063,3.269)	0.433
Smoking habits, n (%)					
NO				Reference	
YES	-0.155	0.293	0.529	0.857(0.483,1.520)	0.597
Drinking habits, n (%)					
NO				Reference	
YES	-0.113	0.245	0.459	0.893(0.552,1.445)	0.646
History of hypertension,n(%)					
NO				Reference	
YES	-0.277	0.358	0.774	0.758(0.376,1.529)	0.439
History of diabetes,n(%)					
NO				Reference	
YES	0.535	0.4	1.339	1.708(0.780,3.737)	0.181
History of coronary heart disease, n(%)					
NO				Reference	
YES	-0.468	1.008	0.464	0.626(0.087,4.517)	0.642
BMI, kg/m2	-0.056	0.037	1.503	0.946(0.880,1.017)	0.133
Course of disease,month	-0.015	0.01	1.522	0.985(0.967,1.004)	0.128
Tumor location, n(%)					
Glottic				Reference	
Non-Glottic	0.701	0.242	2.89	2.015(1.253,3.241)	0.004
Degree of differentiation, n (%)					
poorly				Reference	
Moderate	0.39	0.523	0.746	1.477(0.530,4.117)	0.456
Highly	0.008	0.544	0.015	1.008(0.347,2.928)	0.988
pT staging, n(%)					
1				Reference	
2	-1.095	1.163	0.941	0.334(0.034,3.271)	0.346
3	-2.007	1.031	1.947	0.134(0.018,1.014)	0.052
4	-1.392	1.019	1.366	0.249(0.034,1.832)	0.172
AJCC staging, n(%)					
III				Reference	
IV_a_/IV_b_	0.924	0.292	3.162	2.520(1.421,4.469)	0.002
Vascular thrombosis, n (%)					
NO				Reference	
YES	0.653	0.319	2.049	1.922(1.029,3.589)	0.04
Peripheral nerve infiltration, n(%)					
NO				Reference	
YES	0.513	0.359	1.43	1.670(0.827,3.374)	0.153
Striated muscle invasion, n(%)					
NO				Reference	
YES	0.655	0.249	2.627	1.925(1.181,3.137)	0.009
Thyroid cartilage infiltration, n (%)					
NO				Reference	
YES	-0.172	0.243	0.708	0.842(0.523,1.355)	0.479
Surgical margin, n(%)					
Negatives				Reference	
Positive	0.878	0.3	2.924	2.407(1.336,4.336)	0.003
pN stages, n(%)					
0				Reference	
1	0.348	0.378	0.922	1.417(0.676,2.970)	0.356
2	1.041	0.259	4.016	2.831(1.703,4.704)	<0.001
3	-14.825	2714.368	0.005	0.000(0.000,Inf)	0.996
Cervical lymph node metastases n (%)					
NO				Reference	
YES	0.755	0.242	3.124	2.128(1.325,3.418)	0.002
Contralateral cervical lymph node metastasis, n (%)					
NO				Reference	
YES	1.137	0.293	3.875	3.118(1.754,5.542)	<0.001
Total number of lymph nodes cleared (n)	0.006	0.006	0.961	1.006(0.994,1.019)	0.337
Total number of positive lymph nodes (n)	0.158	0.036	4.366	1.171(1.091,1.257)	<0.001
Proportion of positive lymph nodes	1.567	0.494	3.171	4.790(1.819,12.615)	0.002
Type of primary resection, n (%)					
Partial laryngectomy				Reference	
Total laryngectomy	0.108	0.253	0.425	1.114(0.678,1.830)	0.671
Side of cervical lymph node dissection, n(%)					
NO				Reference	
Unilateral	-0.686	0.35	1.96	0.504(0.254,1.000)	0.05
Bilateral	-0.05	0.308	0.164	0.951(0.520,1.740)	0.87
Postoperative radiotherapy, n (%)					
NO				Reference	
YES	0.146	0.253	0.578	1.158(0.705,1.901)	0.563
Post-operative chemotherapy, n (%)					
NO				Reference	
YES	0.113	0.28	0.403	1.119(0.647,1.937)	0.687
NLR	0.038	0.013	2.998	1.038(1.013,1.064)	0.003
PLR	0.001	0	1.543	1.001(1.000,1.001)	0.123
LMR	-0.107	0.085	1.261	0.899(0.762,1.061)	0.207
SII	0	0	2.305	1.000(1.000,1.000)	0.021
PNI	-0.02	0.006	3.328	0.980(0.969,0.992)	<0.001
ALI	-0.001	0	1.408	0.999(0.999,1.000)	0.159
SIRI	0.02	0.042	0.478	1.020(0.939,1.109)	0.633

### 3.3 Rad-score

**Figure 3:**
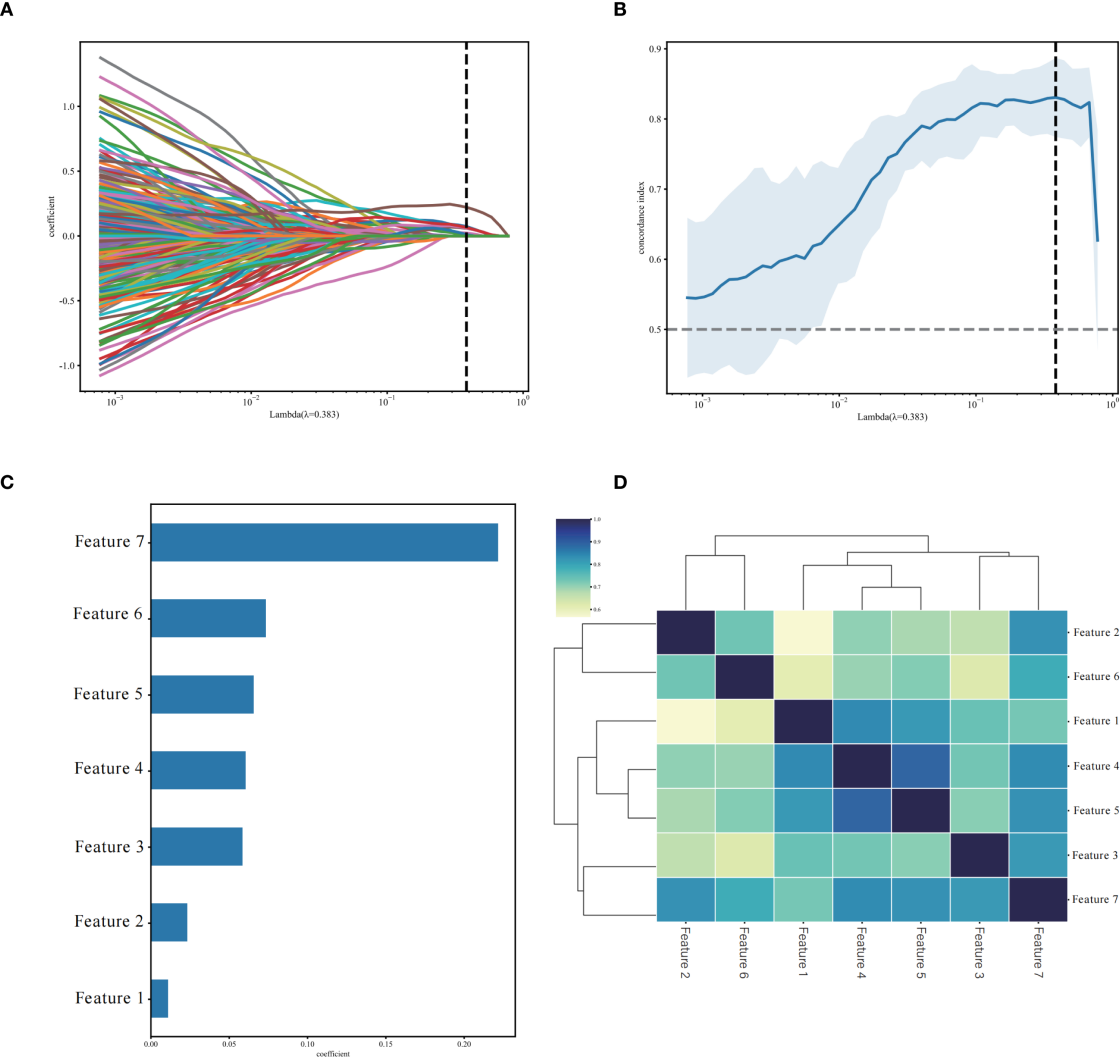
Selection of non-zero coefficient radiomics features using the least absolute shrinkage and selection operator (LASSO) regression model. **(A)** LASSO regularization path diagram; **(B)** C-index coefficient plot using 10-fold cross-validation; **(C)** 7 selected radiomics features and their weight coefficients; **(D)** correlation clustering heatmap of 7 radiomics features.

Through PyRadiomics, 3232 radiomics features were extracted, with 2658 (82.24%) retained after ICC screening, including 10 categories of features such as first-order statistics (360) and Hessian matrix (780) (**Supplementary Figure 1**). Univariate Cox regression analysis further screened 413 significant features (p< 0.05), and LASSO ultimately retained seven non-zero coefficient features, including one first-order statistical feature, one three-dimensional shape feature, three GLCM, one GLRLM, and one GLSZM (**Figure 3**, **Table 2**). The Rad-score was computed as a linear combination of these features (**Equation 1**):

**Table 2:** Seven radiomics signature coefficients selected using LASSO-cox regression.

Variables	Radiomics features	Coefficient
Feature1	wavelet_LLL_glszm_ZoneEntropy	0.010967
Feature2	gradient_firstorder_Skewness	0.023122
Feature3	original_shape_Maximum3DDiameter	0.058509
Feature4	wavelet_HHL_glcm_Idn	0.060522
Feature5	wavelet_HLL_glcm_Idn	0.065519
Feature6	lbp_3D_m2_glrlm_GrayLevelVariance	0.07332
Feature7	wavelet_LLL_glcm_Imc1	0.221575


(1) $$\begin{array}{c} h(t)=h0(t)\exp(0.489\cdot Feature1-0.185\cdot Feature2-0.071\cdot Feature3+0.39\cdot Feature4\\ +0.897\cdot Feature5+0.575\cdot Feature6+0.05\cdot Feature7) \end{array}$$

### 3.4 Fusion model construction

#### 3.4.1 FF-Model

**Figure 4:**
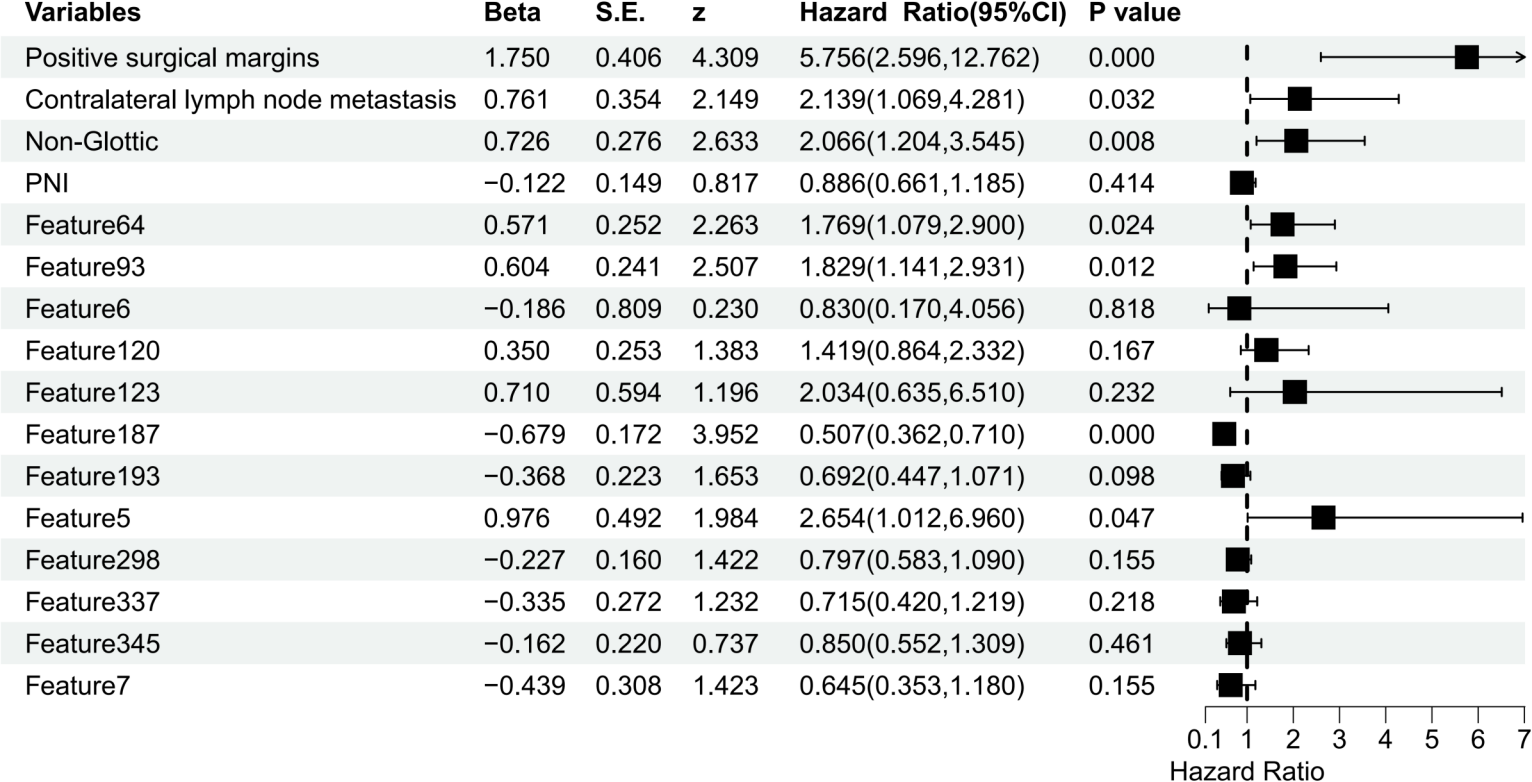
Cox Regression-Based Feature-Level Fusion Model.

To develop the FF-Model, we integrated 13 clinicopathological variables with 413 radiomic features, resulting in a 426-dimensional dataset. Subsequently, LASSO-Cox regression identified 16 significant predictors, including PNI, tumor location, surgical margin status, contralateral cervical lymph node metastasis, and 12 radiomics features (**Supplementary Figure 2**, **Supplementary Table 4**). Multivariate Cox regression identified seven independent predictors of RFS (p< 0.05), among which were positive surgical margins and contralateral lymph node involvement (**Figure 4**).

#### 3.4.2 DF-Model

The DF-Model formula based on multifactorial Cox regression is as follows:


$$h(t)=h_{0}(t)\exp(0.009\cdot Clinic-score+0.036\cdot Rad-score)$$

**Figure 5:**
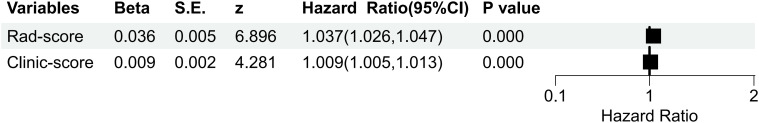
Cox Regression-Based Decision-Level Fusion Model.

**Figure 6:**
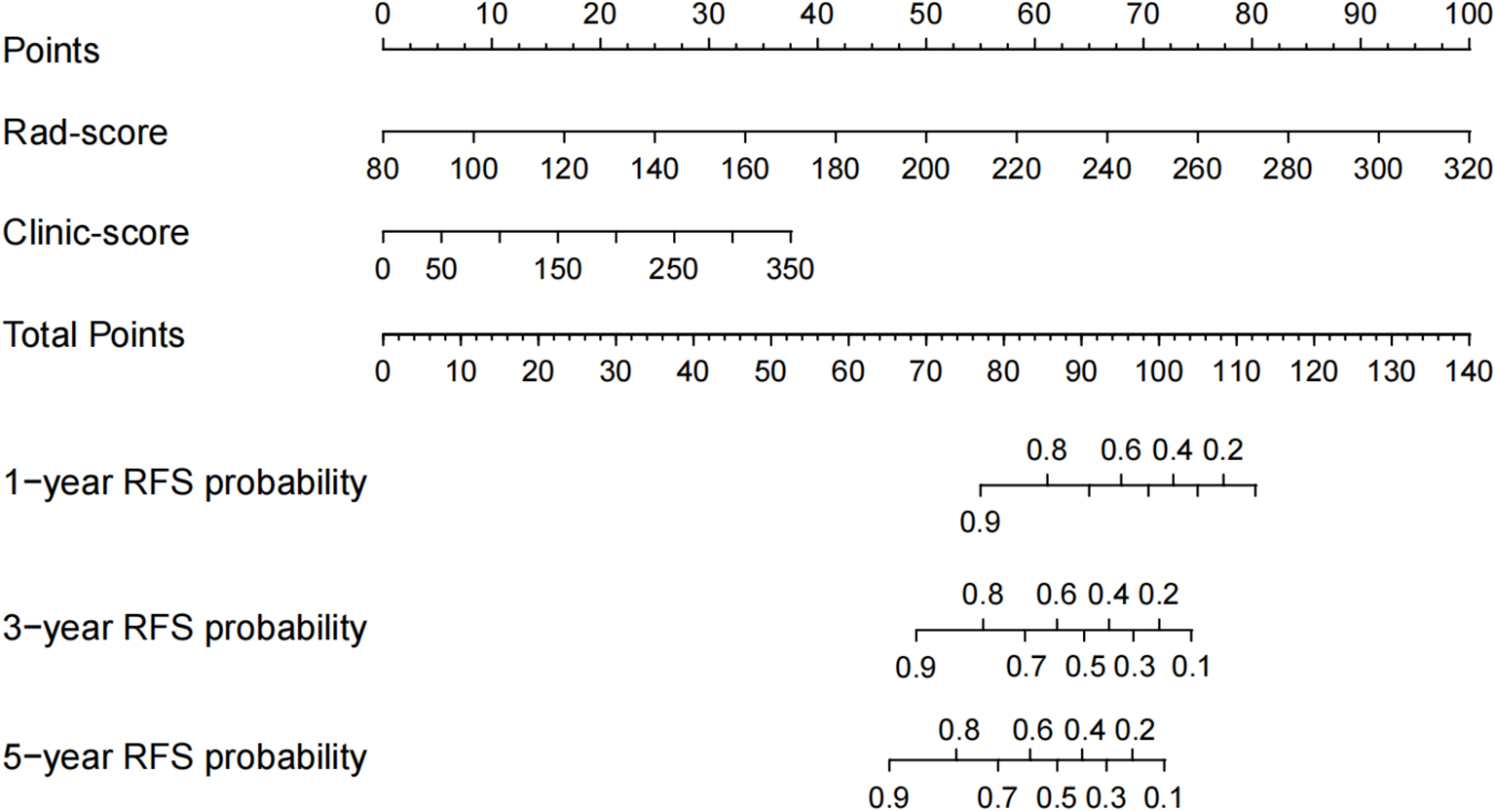
Nomogram Based on a Decision-Level Fusion Strategy.

Both the Clinic-score and Rad-score significantly impacted the postoperative RFS of locally advanced LSCC (p< 0.001; **Figures 5**, **6**).

### 3.5 Model performance comparison

#### 3.5.1 Discrimination

In the training set, the C-index of the DF-Model was 0.847 (95% CI: 0.811–0.884), which was significantly higher than the Clinic-score (0.723; p< 0. 001), Rad-score (0.828; p = 0.099), and AJCC stage (0.608; p< 0.001). The C-index of the FF-Model was 0.878 (95% CI: 0.838–0.917), which was significantly higher than that of the DF-Model (p = 0.024). In the validation set, the DF-Model achieved a C-index of 0.826 (95% CI: 0.763–0.889), showing a statistically significant improvement over the FF-Model (0.741; p = 0.047), Rad-score (0.734; p = 0.033), Clinic-score (0.723; p = 0.002), and AJCC stages (0.58; p< 0.001, **Table 3**).

**Table 3:** Comparison of C-indices among five predictive models.

Models	C-index(95% CI)		*P_1_*		*P_2_*		*P_3_*		*P_4_*	
	Training set	Validation set	Training set	Validation set	Training set	Validation set	Training set	Validation set	Training set	Validation set
DF-Model	0.847(0.811− 0.884)	0.826(0.763− 0.889)	-	-	-	-	-	-	-	-
FF-Model	0.878 (0.838–0.917)	0.741 (0.638–0.844)	0.024	0.047	-	-	-	-	-	-
Rad-score	0.828 (0.787–0.869)	0.734 (0.627–0.841)	0.099	0.033	0.003	0.828	-	-	-	-
Clinic-score	0.723 (0.658–0.788)	0.657 (0.552–0.763)	<0.001	0.002	<0.001	0.292	0.006	0.369	-	-
AJCC stages	0.608(0.556-0.660)	0.580(0.484-0.676)	<0.001	<0.001	<0.001	0.002	<0.001	0.003	<0.001	0.149

*P_1_, P_2_, P_3_*, and *P_4_* denote the results of Z-tests comparing C-index differences between model pairs: *P_1_*: DF-Model vs. FF-Model, Rad-score, Clinic-score, and AJCC stages; *P_2_*: FF-Model vs. Rad-score, Clinic-score, and AJCC stages; *P_3_*: Rad-score vs. Clinic-score and AJCC stages; *P_4_*: Clinic-score vs. AJCC stages.

**Figure 7:**
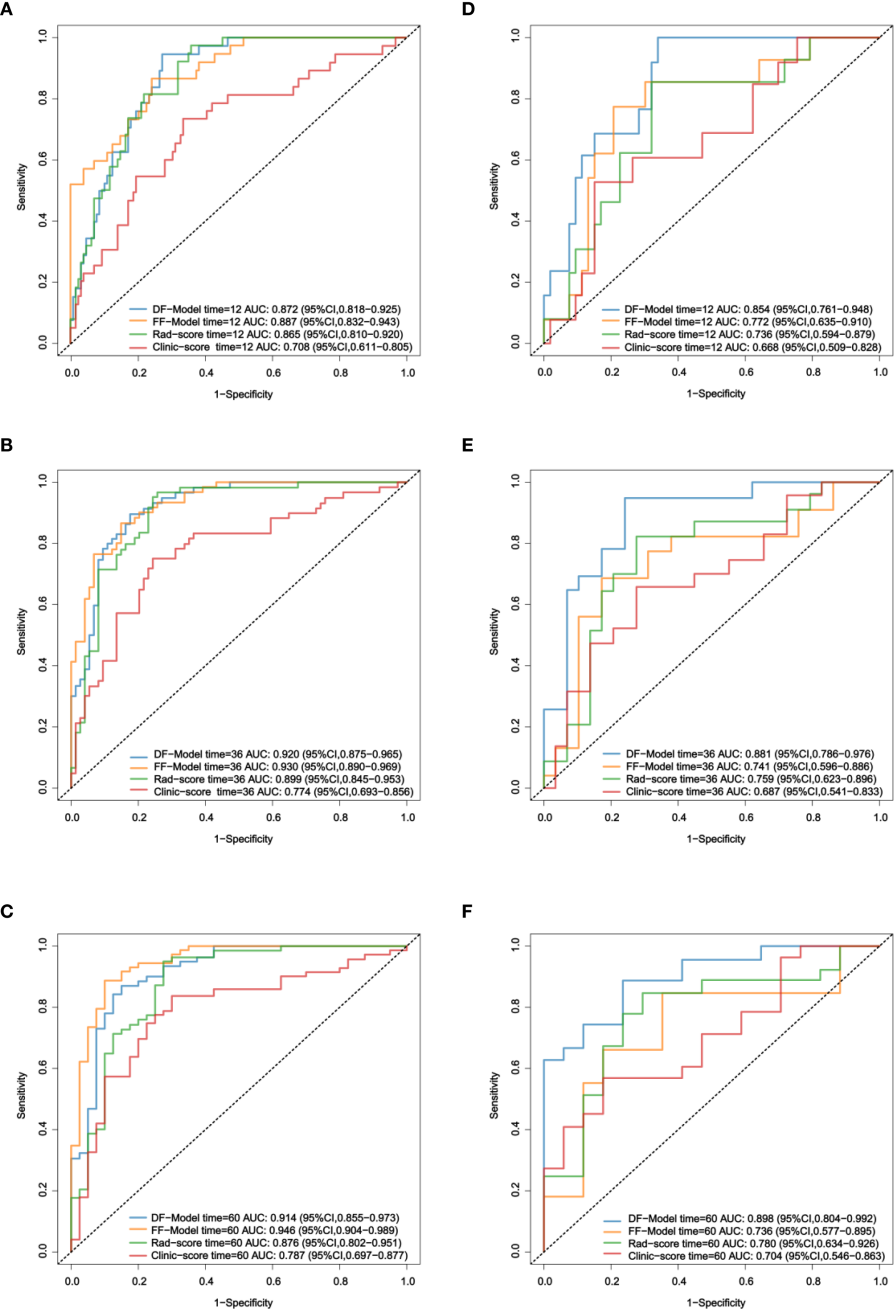
ROC comparison of four models for RFS prediction. **(A–C)** Training set; **(D–F)** Validation set.

The ROC analysis showed no significant difference between the AUC values of the FF-Model and DF-Model in the prediction of 1/3/5-year RFS in the training set (p > 0.05; **Figures 7A–C**). In the validation set, the AUC of the DF-Model was significantly higher than that of the FF-Model for predicting 3-year and 5-year RFS (all p = 0.022). no statistically significant difference was observed in the predictive performance of 1-year RFS across the models (p = 0.206; **Figures 7D–F**).

#### 3.5.2 Calibration

**Figure 8:**
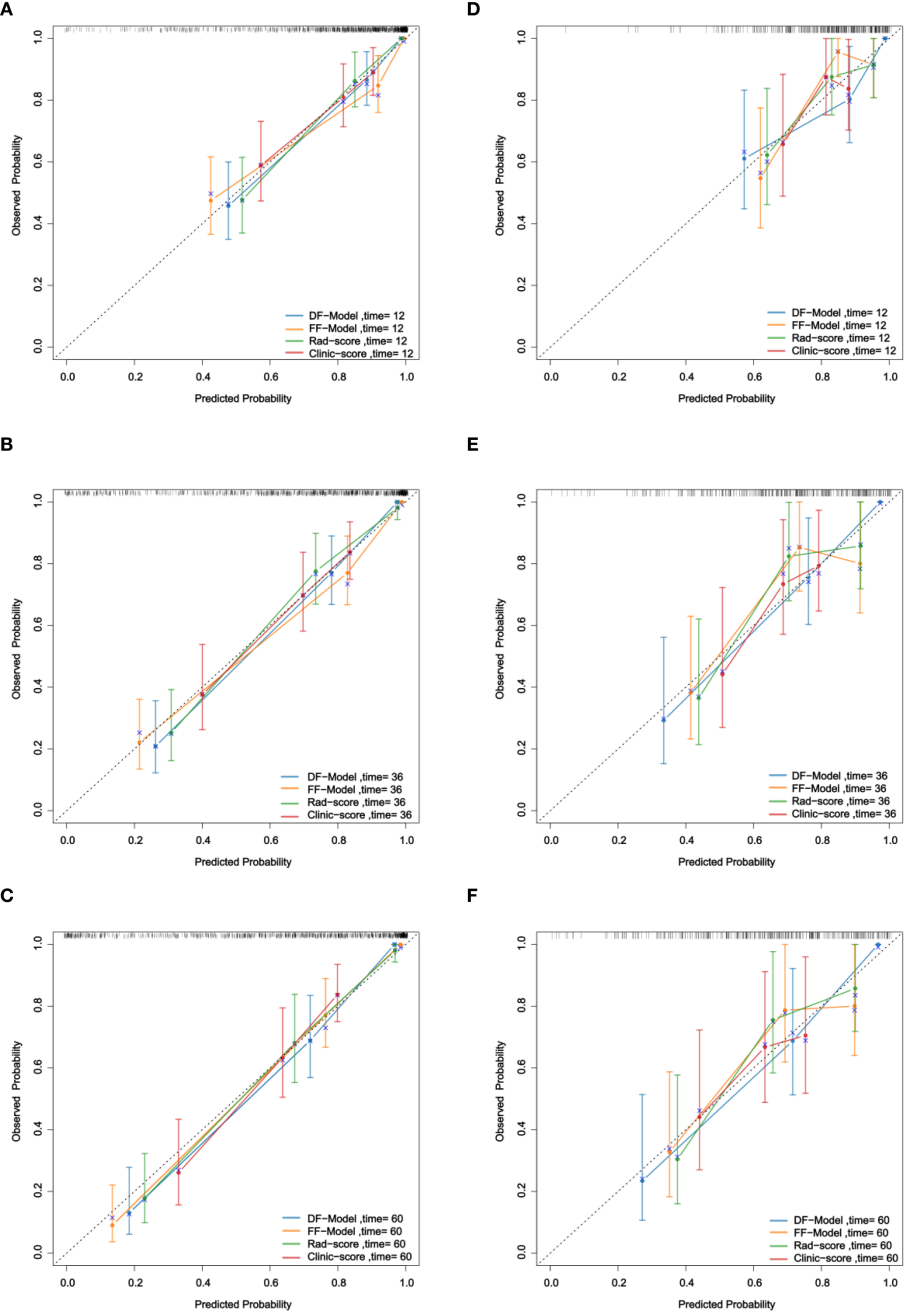
Calibration of four models for RFS prediction. **(A–C)** Training set; **(D–F)** Validation set.

Calibration curve analysis demonstrated that in predicting 1-year, 3-year, and 5-year RFS, the calibration curves of the DF-Model in both the training and validation sets were closer to the ideal diagonal line than those of the FF-Model, Rad-score, and Clinic-score (**Figure 8**), indicating superior calibration performance over the other models.

#### 3.5.3 Clinical utility

**Figure 9:**
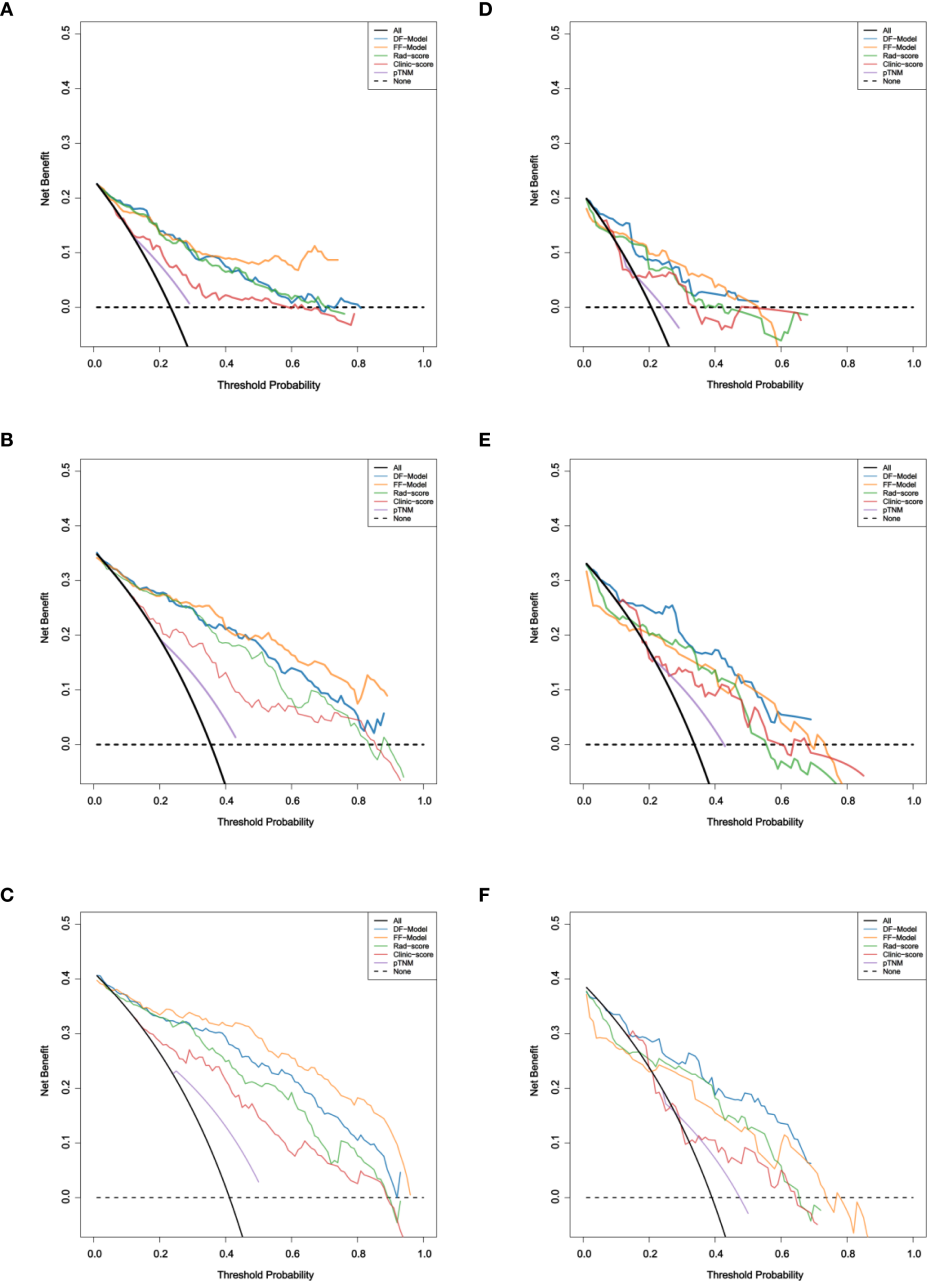
Decision curve analysis of five models for RFS prediction. **(A–C)** Training set; **(D–F)** Validation set.

DCA showed that both fusion models provided greater clinical utility than the single-modality models and the AJCC staging system for predicting 1-, 3-, and 5-year RFS (**Figure 9**).

In the training set, the FF-Model yielded the highest net benefit across most threshold probabilities. In the validation set, the DF-Model generally outperformed other models, particularly for 3- and 5-year predictions. Although the FF-Model showed slightly higher net benefit at some thresholds for 1-year RFS, the DF-Model remained superior to all single-modality models.

#### 3.5.4 cNRI and IDI tests

The DF-Model exhibited better discriminative ability than the single-modal and the FF-Model in both the cNRI and IDI tests. In the 1-year prediction, the cNRI increased by 32.6% (p = 0.040), 51.3% (p = 0.020), and 23.4% (p< 0.001) compared to the FF-Model, Rad-score, and Clinic-score, respectively. Although the IDI gain did not reach statistical significance (p = 0.079), it transcended the minimal clinically important difference (MCID = 5%) (31) and thus, might have potential value. In the 3-year prediction, the cNRI showed significant improvement compared to the Rad-score (51.5%; p = 0.020) and Clinic-score (70.1%; p< 0.001). Additionally, the IDI exhibited superior performance compared to the Clinic-score (27.3%; p = 0.02) and Rad-score (19.1%; p< 0.001). Regarding the 5-year prediction, an even more robust performance was observed, with the cNRI increasing to 67.0% (p< 0.001) compared to the Clinic-score, and 47.8% (p = 0.020) compared to the Rad-score. The IDI exhibited a similar trend (**Table 4**).

**Table 4:** Comparison of the DF-Model with the FF-Model, Rad-score, and Clinic-score using IDI and cNRI metrics.

Model	Time(year)	Datasets	Evaluation indicators	Estimate (95% CI)	p-value
FF-Model	1	Training set	IDI	-0.155 (-0.241, -0.043)	<0.001
			cNRI	-0.511 (-0.655, 0.025)	0.079
		Validation set	IDI	0.093 (-0.004, 0.182)	0.079
			cNRI	0.326 (0.008, 0.546)	0.04
Rad-score		Training set	IDI	0.037 (-0.047, 0.100)	0.455
			cNRI	0.264 (-0.318, 0.481)	0.436
		Validation set	IDI	0.115 (-0.023, 0.186)	0.079
			cNRI	0.513 (0.091, 0.766)	0.02
Clinic-score		Training set	IDI	0.159 (0.068, 0.236)	<0.001
			cNRI	0.433 (0.234, 0.606)	<0.001
		Validation set	IDI	0.173 (0.038, 0.311)	<0.001
			cNRI	0.234 (0.060, 0.668)	<0.001
FF-Model	3	Training set	IDI	-0.087 (-0.153, -0.019)	0.02
			cNRI	-0.530 (-0.660, 0.084)	0.079
		Validation set	IDI	0.143 (-0.041, 0.287)	0.099
			cNRI	0.290 (-0.026, 0.662)	0.059
Rad-score		Training set	IDI	0.058 (-0.025, 0.115)	0.158
			cNRI	0.207 (-0.403, 0.551)	0.851
		Validation set	IDI	0.191 (0.044, 0.288)	<0.001
			cNRI	0.515 (0.075, 0.742)	0.02
Clinic-score		Training set	IDI	0.254 (0.150, 0.337)	<0.001
			cNRI	0.570 (0.386, 0.732)	<0.001
		Validation set	IDI	0.273 (0.065, 0.418)	0.02
			cNRI	0.701 (0.296, 0.852)	<0.001
FF-Model	5	Training set	IDI	-0.116 (-0.184, -0.037)	<0.001
			cNRI	-0.584 (-0.736, 0.000)	0.04
		Validation set	IDI	0.158 (-0.022, 0.327)	0.079
			cNRI	0.296 (-0.075, 0.733)	0.119
Rad-score		Training set	IDI	0.073 (-0.013, 0.124)	0.079
			cNRI	0.105 (-0.431, 0.663)	0.832
		Validation set	IDI	0.170 (0.024, 0.317)	0.02
			cNRI	0.478 (0.049, 0.681)	0.02
Clinic-score		Training set	IDI	0.254 (0.150, 0.337)	<0.001
			cNRI	0.570 (0.386, 0.732)	<0.001
		Validation set	IDI	0.305 (0.128, 0.465)	<0.001
			cNRI	0.670 (0.311, 0.822)	<0.001

#### 3.5.5 Subgroup analysis

**Figure 10:**
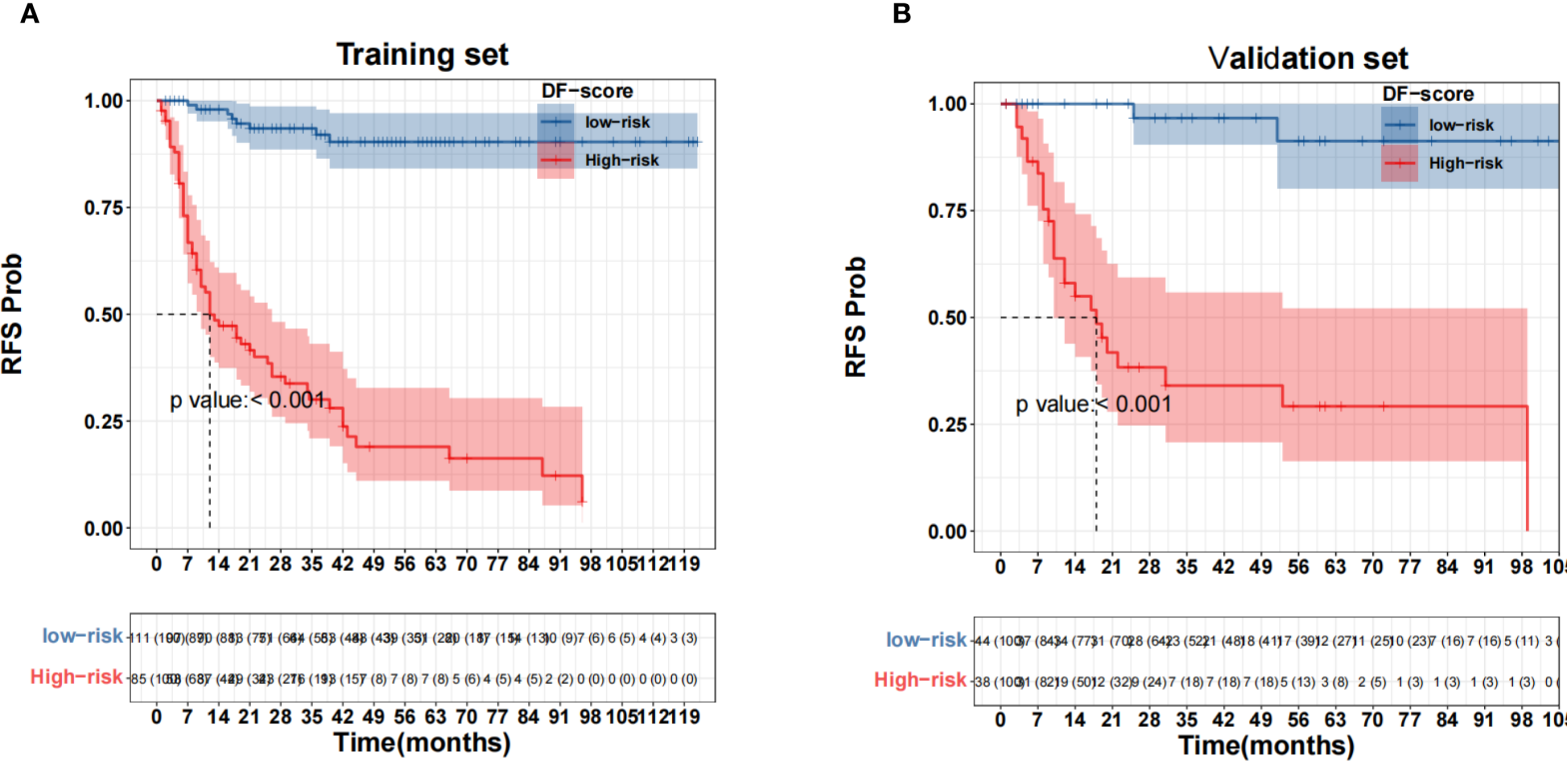
Survival analysis using the fusion model threshold (81.1). **(A)** Training set; **(B)** Validation set.

**Figure 11:**
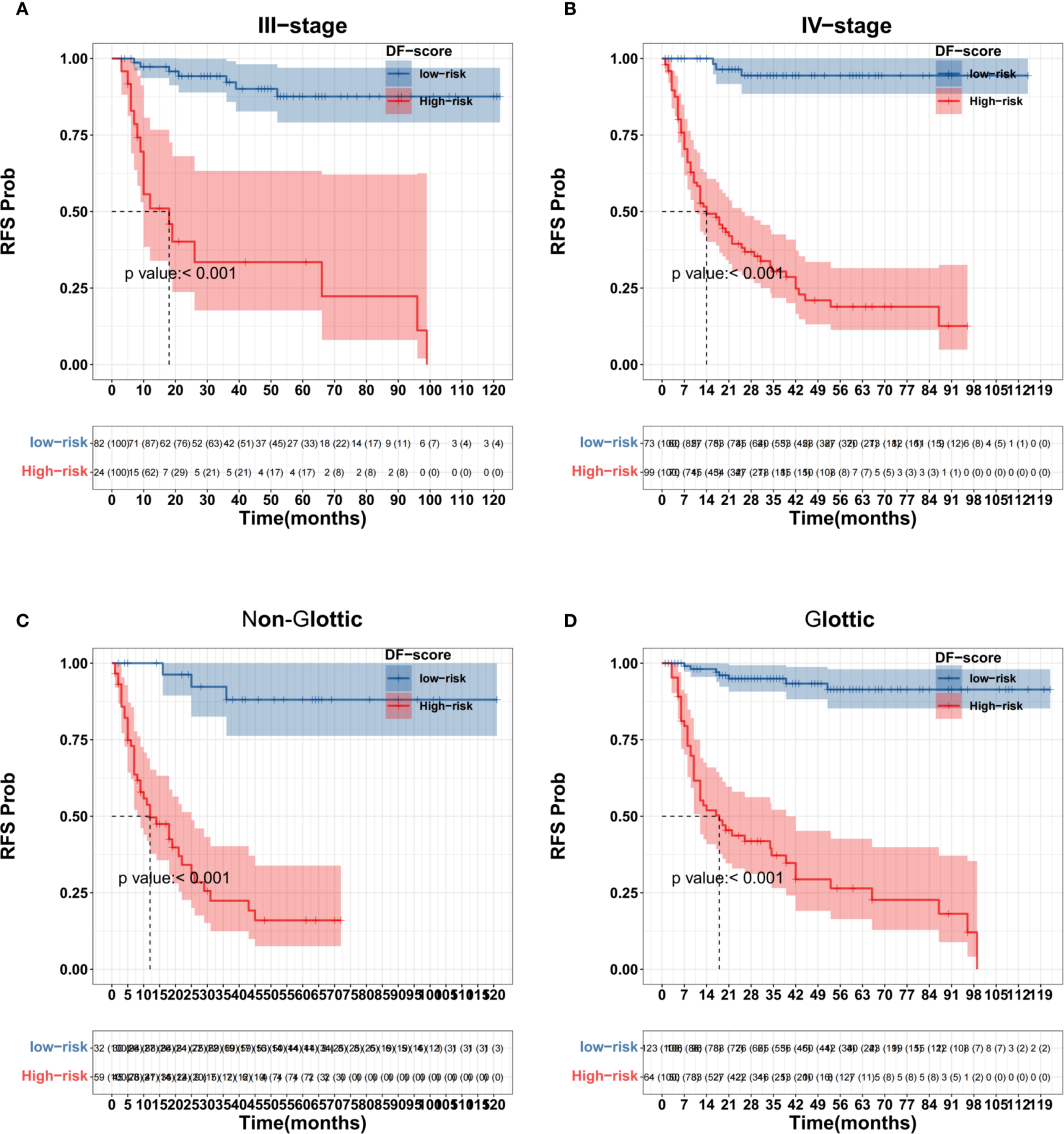
Subgroup survival analysis using the DF-Model threshold (81.1). **(A)** Stage III; **(B)** Stage IVa/IVb; **(C)** Non-glottic; **(D)** Glottic.

X-tile software was used to determine the cutoff value of the DF-Model, which was 81.1, to divide the training set into low-risk (<81.1) and high-risk (≥81.1) subgroups. Kaplan-Meier analysis showed significantly worse recurrence-free survival (RFS) in the high-risk group compared to the low-risk group in both the training and validation cohorts (all p< 0.001; **Figure 10**). The prognostic value of the DF-Model remained significant across subgroups defined by AJCC stage (III vs. IV) and tumor location (glottic vs. non-glottic) (all p< 0.001; **Figure 11**).

## 4 Discussion

### 4.1 State of the art in multimodal data fusion applications

Locally advanced LSCC has a high postoperative recurrence rate, and the traditional AJCC staging system is not sufficient for precise prognosis prediction, making it difficult to develop personalized treatment plans. In this case, multimodal data fusion provides a different method for improving prediction effectiveness. Different data modalities complementarily reflect the biological behavior of tumors. Macroscopic invasiveness (such as rhabdomyolysis invasion and lymph node metastasis burden) and preoperative blood markers (such as PNI) characterize the host’s systemic inflammatory and nutritional status, whereas radiomic features (such as wavelet transform texture and gray entropy) quantify the heterogeneity of the tumor microenvironment. Studies have attempted to integrate multimodal data to improve predictive efficacy in head and neck squamous cell carcinoma (HNSCC). For example, Tseng et al. (32) integrated clinical, pathological, and genetic variation data to construct an elastic net Cox model to predict the survival risk in patients with oral cancer. Wang et al. (33) integrated radiomic and pathomic features to develop a Particle Swarm Optimization–Support Vector Machine model aimed at assessing the response to neoadjuvant chemotherapy among individuals diagnosed with nasopharyngeal carcinoma. Yin et al. (34) integrated multiple immuno-omics data to develop a Computational Model for Predicting Immunotherapy Response model for identifying patient populations sensitive to immunotherapy and chemotherapy. Cavalieri et al. (35) established a large-scale multiomics database, BD2Decide, to provide a rich data resource for head and neck cancer research. Additionally, studies have enhanced model performance by integrating radiomic features from different imaging modalities. For instance, Tomita et al. (36) utilized radiomic data obtained from multiple magnetic resonance imaging (MRI) scans to develop a deep learning model designed to predict the likelihood of 2-year progression-free survival (PFS) in patients with laryngeal and hypopharyngeal malignancies. Lin et al. (37) constructed a multimodal radiomics scoring model based on preoperative MRI images of patients with advanced sinonasal squamous cell carcinoma to predict early disease recurrence. Huynh et al. (38) evaluated the performance of traditional radiomics versus convolutional neural network (CNN) models in forecasting patient survival outcomes in HNSCC, and found that the predictive capability of CNN models improved when combined with clinical and radiomic features. Li et al. (39) developed multiple prediction models and demonstrated that the DL-Model achieved higher AUC, accuracy, and specificity in the validation set. Wang et al. (40) also showed that the DL-Model based on multimodal radiomic features achieved the highest AUC values (0.89–0.90) across all datasets, along with optimal sensitivity (82–88%) and specificity (79–85%).

### 4.2 Core findings and comparison with other studies

In the field of laryngeal cancer prognostic prediction, previous studies have mainly focused on single-modality data or small patient cohorts. Zhong et al. (25) used positron emission tomography (PET)-CT metabolic parameters to construct a random forest model to predict disease progression; Choi et al. (41) combined radiomic scores with clinical variables, which increased the C-index of survival prediction to 0.958; Lin et al. (42) found that intra-tumoral/peri-tumor features during the mid-radiation process performed better than pre-radiation models in prediction; Agarwal et al. (43) used pre-treatment CT image data of 60 patients treated with chemoradiotherapy and found that the entropy of medium-filtered texture features is an independent predictor. However, these studies have issues such as incomplete selection of regions of interest and some cases of hypopharyngeal cancer, which makes it difficult to apply these findings elsewhere. Al-Ibraheem et al. (44) used PET/CT data from 68 patients with laryngeal cancer to build a Cox model demonstrating that total lesion glycolysis and metabolic tumor volume have independent predictive values for PFS. Nakajo et al. (45) developed an RSF model using PET/CT features of 49 patients to predict PFS. Chen et al. (24) built a radiomics nomogram with a C-index of 0.913 using CT data from 136 cases of LSCC; however, these studies included early stage laryngeal cancer, which could add some selective bias. Rajgor et al. (26) studied 72 patients with advanced laryngeal cancer, using shape compactness and gray-level zone length matrix−gray-level nonuniformity modeling to predict 5-year survival, achieving a C-index of 0.759, outperforming the clinical model’s C-index of 0.655. Nevertheless, this investigation was conducted at a single institution and involved a limited number of participants; furthermore, its findings have yet to undergo external validation.

In contrast, this study provided an explicit definition and included 278 individuals with locally advanced LSCC, targeting the prediction of prognosis within this high-risk group of patients. Additionally, we included clinicopathological features such as surgical margin status and the ratio of positive lymph nodes, pre-PNI, and CT radiomics features to capture complementary information. The preoperative PNI indicates the patient’s nutritional and immune status, striated muscle invasion represents tumor aggressiveness, the number of positive nodes represents the extent of tumor metastasis, margin status reflects operation radicality, and radiomics features further quantify tumor microenvironment heterogeneity; therefore, the model enables a comprehensive, multidimensional evaluation of the “tumor characteristics- host status- treatment” framework. This represents a shift beyond the conventional AJCC staging system, which was not designed to predict recurrence at the individual patient level and primarily reflects anatomical extent rather than underlying biological behavior.

The radiomic model incorporated features reflecting diverse aspects of tumor phenotype. Tumor size was represented by the three-dimensional maximum diameter (original_shape_Maximum3DDiameter), with larger values generally associated with more advanced disease and poorer prognosis (47). Tumor intensity distribution asymmetry was captured by first-order skewness (gradient_firstorder_Skewness), where high positive values indicate a more aggressive and heterogeneous tumor phenotype (43). Tumor heterogeneity was further quantified using wavelet-based texture features (e.g., wavelet_LLL_glszm_ZoneEntropy), which measure gray-level inhomogeneity; higher values suggest greater internal complexity and are linked to adverse outcomes (43). Notably, four of the seven radiomic features in the final model were derived from wavelet transformation, underscoring the importance of capturing intratumoral heterogeneity in prognostic prediction (46).

In the present study, the DF-Model achieved a C-index of 0.826 (95% CI: 0.763–0.889) in validation, exceeding the FF-Model (0.741), Rad-score (0.734), and Clinic-score (0.657). Furthermore, it exhibited superior performance in calibration curve analysis and DCA. The cNRI and IDI metrics indicated that the DF-Model demonstrated superior performance in forecasting RFS at 1-, 3-, and 5-year intervals compared to alternative models. In particular, the cNRI enhancement for the 1-year prediction relative to the FF-Model reached 32.6% (p< 0.05). Although the improvements in the 3–5-year predictions did not achieve statistical significance, they exceeded the MCID and may hold clinical value in high-risk recurrence cases. The improving effect of the DF-Model compared with the FF-Model diminishes over time, aligning with the clinical observation that the likelihood of recurrence in locally advanced LSCC decreases with time.

Compared with AJCC staging (training set C-index: 0.608; validation set C-index: 0.58), the two fusion models constructed in this study, the single Rad-score and the single Clinic-score, demonstrated significant advantages in terms of discriminative ability and clinical utility. The DF-Model exhibited robust risk stratification capabilities across different datasets, AJCC staging, and tumor location subgroups (log-rank p< 0.001). For instance, in the stage III subgroup, the DF-Model achieved a significant difference in 3-year RFS (high-risk group: 31% vs. low-risk group: 89%), which could not be achieved by AJCC staging alone. This result indicates that the DF-Model transcends the limitations of anatomical staging and can identify truly low-risk groups among patients with advanced disease. Moreover, DCA results indicate that the DF-Model yields a greater net benefit over most of the clinically relevant threshold probability ranges. However, in the 1-year RFS prediction (threshold probability 0.18–0.4), the FF-Model is slightly superior, possibly because short-term recurrence risk is more dependent on the quality of treatment (such as surgical margin status), whereas medium- and long-term recurrence is driven by tumor heterogeneity and the immune microenvironment.

The enhanced risk stratification capability of the DF-Model holds significant clinical interpretability and direct implications for postoperative management. By accurately identifying high-risk patients beyond conventional staging, clinicians can consider intensifying adjuvant therapy—such as adding chemotherapy to radiotherapy or extending radiation fields—for those most likely to benefit, potentially improving survival outcomes. Conversely, low-risk patients identified by the DF-Model may be candidates for de-escalated treatment or less frequent follow-up, thereby reducing the burden of overtreatment, minimizing long-term toxicities (e.g., dysphagia, xerostomia, voice deterioration), and preserving quality of life. This ability to refine risk assessment within the same AJCC stage (e.g., distinguishing high- from low-risk Stage III patients) enables a more biologically driven, personalized approach to postoperative care, moving beyond anatomical staging alone.

### 4.3 Efficacy difference mechanism of multimodal fusion strategy

The differences in the results of the two fusion strategies can be attributed to their inherent characteristics. At the feature level, feature-level fusion is obtained by combining raw features directly before modeling, so the “information dilution effect” and “interaction noise interference” occur (48). The substantial number of radiomic features (413) compared to clinical feature data (13) suggests a high likelihood of overshadowing certain clinical information, such as the positive ratio of lymphatic metastasis, during the process of dimensionality reduction, which can adversely affect model performance. This phenomenon is evident in the results obtained from applying the FF-Model to the validation set, which demonstrated a decrease in the C-index by Δ = 0.137. These findings challenge the assertion that “fusion is always better than single-modality.”

The DF-Model combines the prediction probabilities or scores of individual modality models to build an ensemble model that preserves independent prediction information (49). In the DF-Model, both the Clinic-score and Rad-score are independent predictors of RFS. Analysis of β-values indicated that 80% of the DF-Model’s reliance is on the Rad-score, suggesting that tumor microenvironment heterogeneity significantly influences recurrence, whereas clinicopathological features act as supplementary factors. Patients misclassified as low-risk by the clinical model yet flagged as high-risk by the radiomic model can be accurately recognized by the DF-Model. These patients may benefit from intensive postoperative imaging follow-up, radiotherapy, and immunotherapy. For low-risk patients, the aim is to prevent overtreatment, conserve medical resources, and alleviate the burden of the illness. In addition, the DF-Model eliminates the demand for extensive data preparation by integrating the probable outputs from the Clinic-score and Rad-score, thereby decreasing technical prerequisites and enhancing applicability in resource-limited places, such as primary hospitals. Patients with lower preoperative PNI levels may have improved outcomes with improved nutrition (such as a protein-rich diet supplemented with ω-3 fatty acids). Further studies are required to verify this hypothesis.

### 4.4 Limitations and future directions

Several limitations should be acknowledged in this research, such as a limited participant count (n = 278), reliance on single-center data, and the absence of external validation to assess generalizability across different clinical environments. The strategies for feature selection and integration may require further refinement, as there is a risk of losing important features. Both feature-level and decision-level fusion may not fully optimize data utilization, leading to predictive uncertainty. Promoting the model is difficult because the model’s complexity and the cost associated with CT radiomics feature extraction hinder its clinical translation.

To facilitate the transition from research to clinical practice, essential next steps should prioritize multicenter prospective validation to confirm generalizability, followed by prospective clinical trials designed to evaluate whether model-guided management leads to tangible improvements in patient outcomes—such as recurrence, survival, and quality of life. Concurrently, integrating the model with established molecular biomarkers, including PD-L1 and HPV status, could refine risk stratification, while the development of an accessible web-based calculator would support real-time, bedside decision-making. Beyond these immediate translational priorities, future research should explore advanced feature engineering methods such as transformer networks, the integration of multiomics data—including genomics and proteomics—to enhance predictive accuracy, dynamic risk updating based on postoperative follow-up, and strategies for clinician education and seamless integration into routine clinical workflows.

## 5 Conclusion

The multimodal feature fusion model developed according to the decision-level fusion strategy (DF-Model) enhances the prediction of postoperative RFS in locally advanced LSCC. This improvement is primarily attributed to the integration of multimodal features derived from clinical pathology, PNI, and CT radiomics for the construction of the model. Its performance surpassed those of the FF-Model, single-modality model, and traditional AJCC staging. The model displayed robust risk stratification capabilities across various AJCC stages and subgroups according to tumor location. Further investigations are encouraged to emphasize multicenter validation, enhancement of predictive algorithms, and integration into clinical workflows in order to support real-world deployment and individualized therapeutic strategies.

## Data Availability

The raw data supporting the conclusions of this article will be made available by the authors, without undue reservation.
