# Supplementary figures and images for: Development and validation of a multimodal feature fusion-based model for predicting postoperative recurrence-free survival in locally advanced laryngeal squamous cell carcinoma
Source: Front Oncol. 2025 Sep 25;15:1685737. doi: 10.3389/fonc.2025.1685737 (PMC12507578; doi:10.3389/fonc.2025.1685737)

A

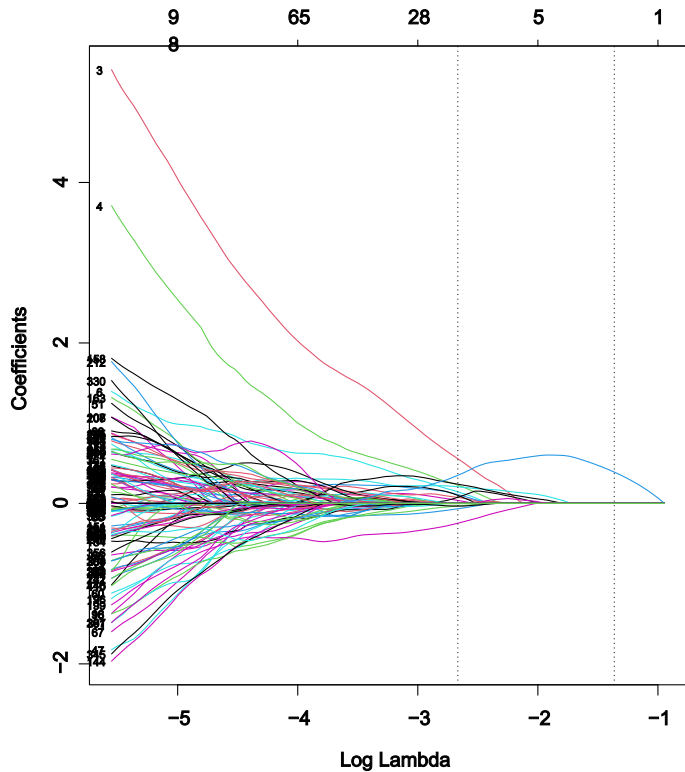

B

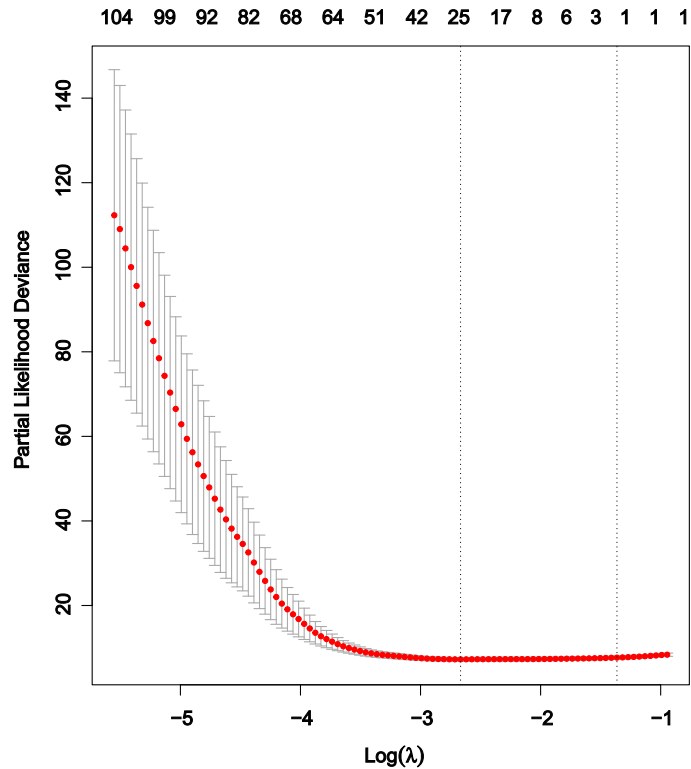

Supplement: Supplementary file 1 [file DataSheet1.zip › updated Datasheet 1/Supplementary files/Supplementary Figure 2: LASSO-Cox–Based Variable Selection for Feature-Level Fusion. (A) Coefficient paths of all variables as a function of the regularization parameter λ. (B) Mean s.pdf]

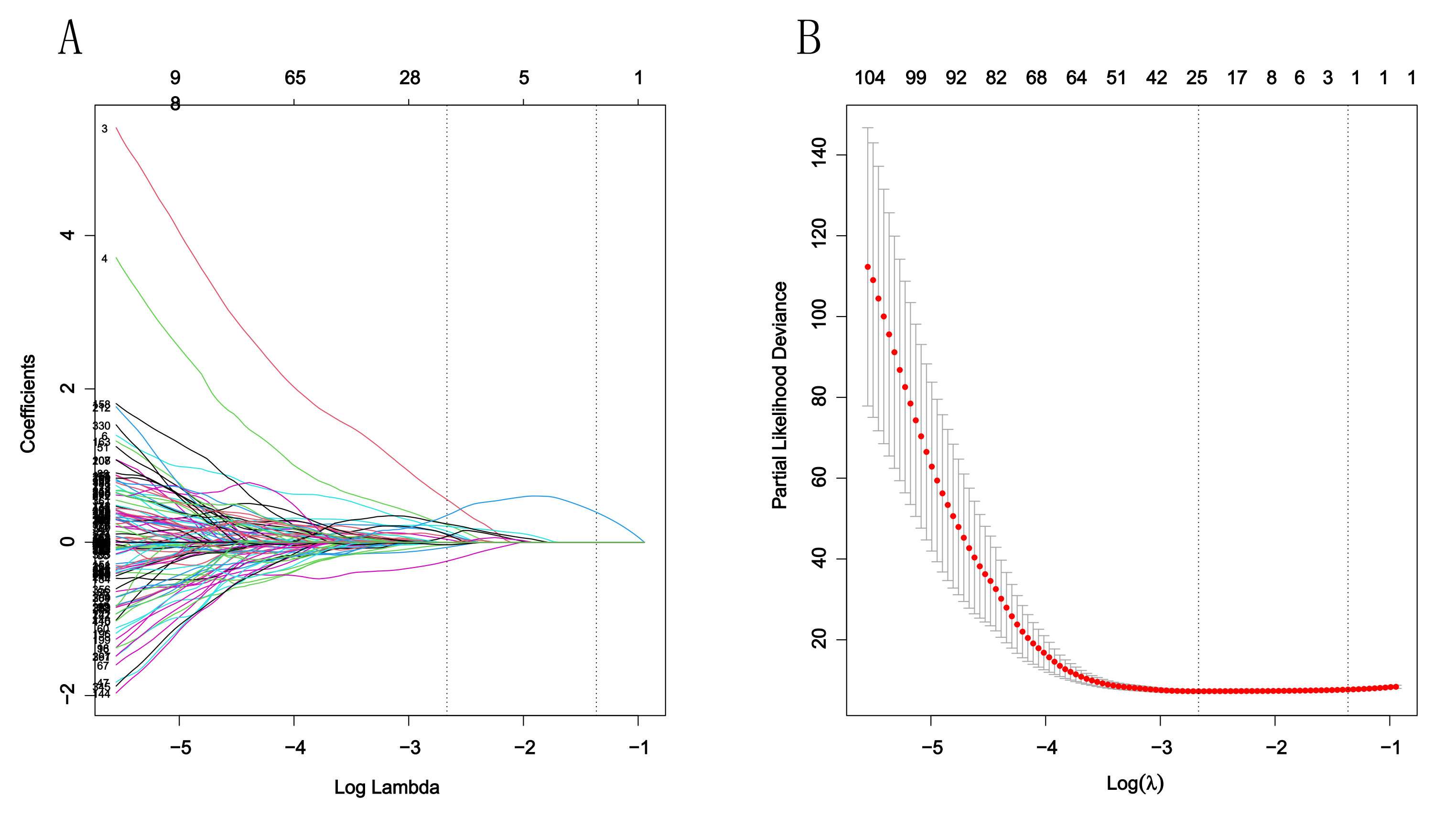

Supplement: Supplementary file 1 [file DataSheet1.zip › updated Datasheet 1/Supplementary files/Supplementary Figure 2: LASSO-Cox–Based Variable Selection for Feature-Level Fusion. (A) Coefficient paths of all variables as a function of the regularization parameter λ. (B) Mean squared error (MSE) plot from 10-f.tif]
